# Structural Study on Hypochlorous Acid-Mediated Chlorination of Betanin and Its Decarboxylated Derivatives from an Anti-Inflammatory *Beta vulgaris* L. Extract

**DOI:** 10.3390/molecules25020378

**Published:** 2020-01-16

**Authors:** Agnieszka Kumorkiewicz-Jamro, Karolina Starzak, Katarzyna Sutor, Boris Nemzer, Zbigniew Pietrzkowski, Łukasz Popenda, Sławomir Wybraniec

**Affiliations:** 1 Department of Analytical Chemistry, Faculty of Chemical Engineering and Technology, Cracow University of Technology, ul. Warszawska 24, 31-155 Cracow, Poland; akumorkiewicz@chemia.pk.edu.pl (A.K.-J.); karolina.starzak@pk.edu.pl (K.S.); ksutor@chemia.pk.edu.pl (K.S.); 2 Chemistry Research, VDF FutureCeuticals, Inc., Momence, IL 60954, USA; bnemzer@futureceuticals.com; 3 Food Science and Human Nutrition, University of Illinois at Urbana-Champaign, Urbana, IL 61801, USA; 4 FutureCeuticals, Inc., 23 Peters Canyon Rd, Irvine, CA 92606, USA; zb@futureceuticals.com; 5 NanoBioMedical Centre, Adam Mickiewicz University in Poznan, Wszechnicy Piastowskiej 3, 61-614 Poznan, Poland; lukasz.popenda@amu.edu.pl

**Keywords:** betacyanins, betalain rich extract, IT-TOF, NMR, inflammation, hypochlorous acid

## Abstract

Hypochlorous acid (HOCl) produced by neutrophils is a part of the natural innate immune response system in the human body, but excessive levels of HOCl can ultimately be detrimental to health. Recent reports suggest that betacyanin plant pigments can act as potent scavengers of inflammatory factors and are notably effective against HOCl. In this contribution, chlorination mechanism and position of the electrophilic substitution in betacyanins was studied by high-resolution mass spectrometry and further structural analyses by NMR techniques, which completed the identification of the chlorinated betacyanins. For the study on the influence of the position of decarboxylation on the chlorination mechanism, a comparison of the chlorination position between betanin as well as 17-, and 2,17-decarboxylated betanins was performed. The structural study confirmed that the chlorination position in betanin occurs within the dihydropyridinic moiety at carbon C-18. Therefore, out of the aqueous free chlorine equilibrium species: HOCl, OCl^−^, Cl_2_, and Cl_2_O, the most potent chlorinating agents are HOCl and Cl_2_O postulated previously and the attack of the Cl^⁺^ ion on the carbon C-18 with a cyclic intermediate version is considered.

## 1. Introduction

Betalains are water-soluble natural plant pigments that occur in most families of the Caryophyllales order [1,2,3,4]. Besides being extensively used in the food industry as natural food colorants, betalains exhibit strong antioxidant and chemopreventive activities [1,2,3,4]. These characteristics contribute to the increased interest in betacyanins, especially those present in *Beta vulgaris* L. root, in oncology applications [3,4]. Furthermore, recent studies indicate that betalains can act as potent scavengers of inflammatory factors [5,6] and may improve various health conditions related to inflammation [7,8].

Hypochlorous acid (HOCl) is an important reactive oxygen species (ROS) that causes oxidation and chlorination reactions. It is produced by activated neutrophils and monocytes via the reaction of H_2_O_2_ with Cl^−^ ions catalyzed by the heme enzyme myeloperoxidase [9,10]. In living systems, HOCl can react at the molecular level with primary amines and other *N*-compounds leading to a formation of chloramines and N-Cl derivatives [11]. The presence of HOCl and its derivatives in intercellular spaces can lead to the local irritation of epithelial tissue cells, damage of proteins, nucleotides, DNA, RNA, fatty acids or cholesterol [11,12,13,14,15].

Recently a novel betalain-rich extract/concentrate (BRE) from the roots of *B. vulgaris* was tested in a pilot clinical study. A short-term treatment with BRE improved the function and comfort of knee joints in individuals with knee distress [7,8]. Said BRE was designed to deliver the benefits of betalains, without the accompanying sugars, nitrates, and calories from the beetroot [16]. During extraction, the naturally occurring sugar derivatives and nitrates are selectively removed until a minimum mass content of 25% betalains remains. BRE contains mainly betanin, neobetanin as well as their decarboxylated derivatives and it is currently used as a dietary supplement [16].

The aim of this contribution was to prove a hypothesis on betacyanin chlorination mechanism by HOCl [5,6,17,18,19,20]. This was accomplished by high-resolution mass spectrometry (ion-trap and time-of-flight mass spectrometry) coupled with high-performance liquid chromatography (LCMS-IT-TOF) and subsequently NMR analyses of resulting chlorination products.

## 2. Results and Discussion

**Figure 1:**
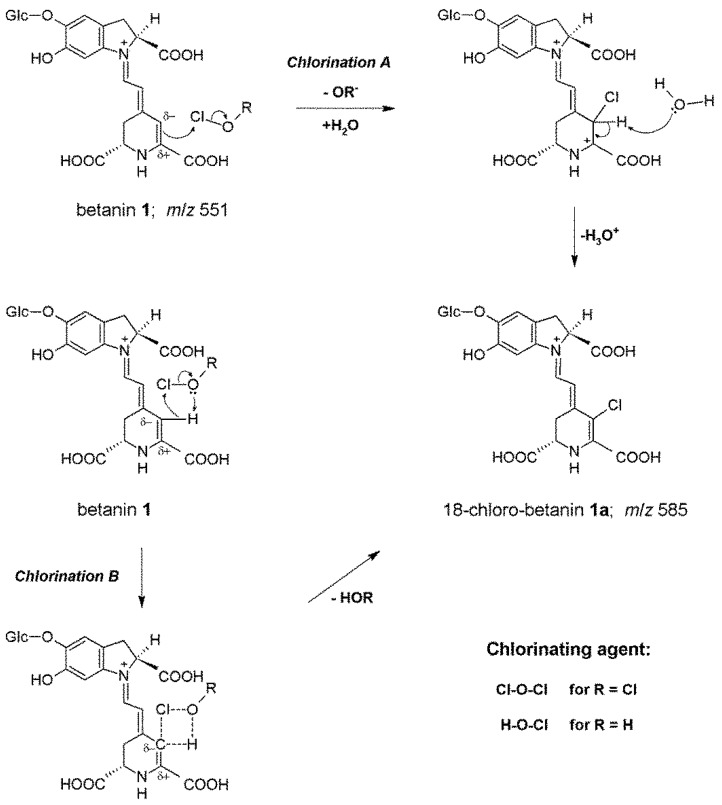
Proposed mechanism of betanin chlorination based on two versions, A and B [5,18], of the Cl^+^ ion attack on the carbon C-18. In version B, a cyclic intermediate [18] formed during electrophilic attack of the whole HOCl or Cl_2_O molecules with the stabilizing effect of the hydrogen bonding is presented.

Our previous preliminary studies on betacyanin chlorination mechanism (*Chlorination A*, Figure 1) indicated a formation of mono-chlorinated betanin and betanidin as well as their decarboxylated derivatives (Table 1) as a result of betacyanin reaction not only with HOCl but also with Cl_2_O which is a potent chlorinating agent and co-exists with HOCl in equilibrium especially in acidic conditions [5,6,18]. Those experiments were monitored by HPLC with low-resolution mass spectrometric detection. The chemical changes were confronted with enzymatic chlorination of betacyanins catalyzed by MPO at NaCl concentration (150 mM) typical to the physiological levels enabling a slow and continuous production of HOCl from Cl^−^ [5].

In this contribution, we performed semi-synthesis of chlorinated betacyanins (Table 1) at a preparative scale for the elucidation of the chlorination position in betacyanins by NMR and finally to confirm the chlorination mechanism. Chlorination of betacyanins is possible in the MPO-enzymatic system even at pH 7.4 [5] and it is vigorous at pH close to 5 corresponding to high inflammation conditions [5,10]. We explored all the pH range (3–7.4) extensively in our previous report [5] on NaOCl and MPO-mediated chlorination of betacyanins and it is evident that for efficient semi-synthesis of chlorinated betacyanins, it is necessary to perform the preparative reaction at pH close to 3 with the use of NaOCl as reagent to obtain sufficient quantities of the products.

**Figure 2:**
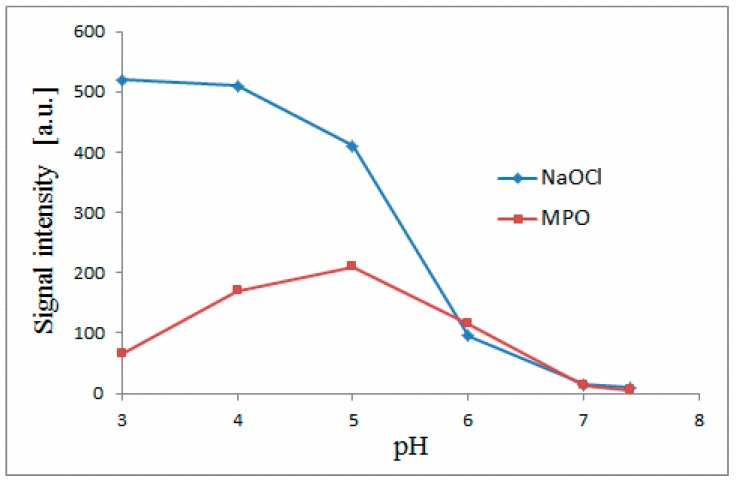
Betanin chlorination activities of the chemical (NaOCl) and enzymatic (MPO + H_2_O_2_ + Cl^−^) systems obtained by LC-MS quantification of generated chlorinated betanin **1a** in dependence on pH in each tested system.

**Figure 3:**
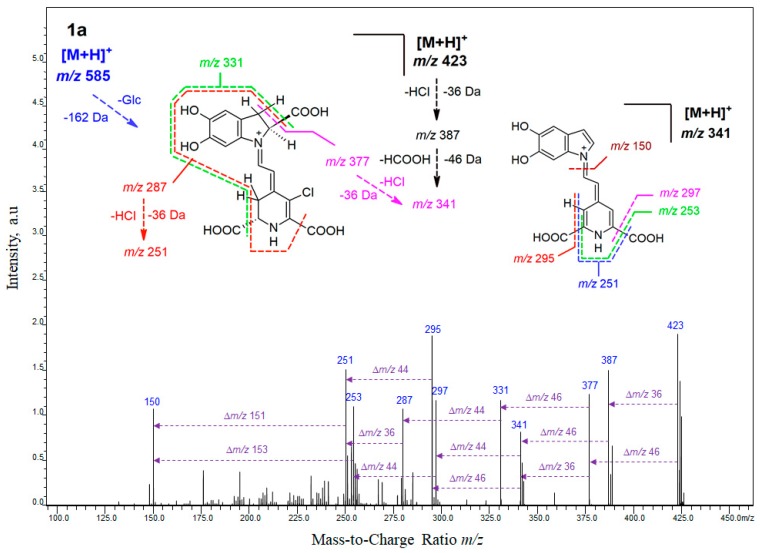
The HRMS fragmentation spectrum obtained by IT-TOF for chlorinated betanin **1a** and chlorinated decarboxylated derivatives **2a** and **6a** as well as fragmentation pathways for their deglucosylated protonated ions.

In Figure 2, the activities of the chemical (NaOCl) and enzymatic (MPO + H_2_O_2_ + Cl^−^) chlorination systems are presented in the form of abundance of generated chlorinated betanin **1a** depending on pH. The continuity of this activity over the whole tested pH range is very well represented. It should be noticed that the activity of the MPO system is also detected at the non-physiological pH range (3-4). Therefore, the results of this structural study obtained at pH 3 are related to the beneficial effect of betacyanins in scavenging of HOCl and are in agreement with the MPO activity that is expressed in both the physiological and non-physiological pH ranges. The continuity of this activity over the whole tested pH range confirms the above-mentioned conclusion which is supported further by the identity of the chromatographic peaks of obtained chlorinated betacyanins. At each pH, the identity of a given chlorinated betacyanin is this same (the same retention time, molecular formula as well as MS-fragmentation pattern (Table 2, Figure 3)), therefore, the chlorinated betacyanins that were structurally determined in this study are identical to the chlorinated betacyanins obtained at physiological pH ranges in this and previous study [5].

In the first stage of this study, detailed mass spectrometric experiments on molecular formulas of chlorinated pigments were performed by the high-resolution IT-TOF technique (Table 2) and were supported by elucidation of their ion fragmentation pathways (Figure 3 and Figure S2 in Supplementary Materials).

Subsequently, the position of the electrophilic substitution in betacyanins and the chlorination mechanism were structurally elucidated by NMR techniques. The reactions (Figure 1) with HOCl and Cl_2_O are proposed according to an electrophilic mechanism based upon leaving group ability from Cl^+^ in HOCl (-OH^−^) and in Cl_2_O (-OCl^−^) [17]. Based on the NMR results showing the position of the attack by Cl^+^ on carbon C-18 we can postulate now a general substitution mechanism (version A). This is also supported by an inactivity of neobetanin toward chlorination [5]. Neobetanin (Figure S1), is an aromatized betanin with a pyridine ring resulting from betanin oxidation. The fact that this ring cannot be chlorinated for generation of chlorinated neobetanin suggests that in betacyanins, only the unsaturated bond is attacked, preferably at carbon C-18 because of its partial negative charge [21]. This is in agreement with all ^1^H NMR data obtained for betacyanins in D_2_O confirming the presence of acidic proton H-18 that can be exchanged by deuterium within few hours [21]. Therefore, this proton can be easily exchanged by Cl^+^ as well.

The intriguing lack of betacyanin chlorination by Cl_2_, which we extensively studied [5], confirms that only Cl^+^ leaving from HOCl or Cl_2_O can be the chlorinating factor. In contrast, Cl_2_ is still active as an oxidizing agent [19]. There is a doubt about a possible action of H_2_OCl^+^ as oxidizing species [19,20] but this entity is frequently associated with the chlorination potency as carrying Cl^+^ as a chlorinating factor [19,20], however, this hypothesis has been not confirmed for a few decades [19]. We presented its possible impact on the chlorination of betacyanins previously [5] to underline the Cl^+^ role in the process. In this contribution, we propose another version of the reaction (*Chlorination B*, Figure 1) with a cyclic intermediate [18] formed during the electrophilic attack of the whole HOCl or Cl_2_O molecules as a result of the Cl-O bond polarization (Cl^δ+^-O^δ−^) with the stabilizing effect of the hydrogen bonding. Due to the negative charge on carbon C-18, the nucleophilic attack of the oxygen [18] is not possible. Therefore an oxidation of this carbon did not take place. This was confirmed by our LC-MS analyses of the chlorination products in which possible oxidation products (such as hydroxylated derivatives) were not detected.

For the study on the influence of the position of decarboxylation on the chlorination mechanism, a comparison of the chlorination position between betanin as well as 17-, and 2,17-decarboxylated betanins was performed (Figure 3 and Figure S2). These mass spectrometric studies were supported by further structural analyses by NMR techniques, which completed the identification of the chlorinated betacyanins with results presented in this report (Table 3 and Figure S3). In each case, the chlorination position was established at carbon C-18 confirming the above proposed mechanism of chlorination. Further mass spectrometric results are presented for the chlorinated 2- and 15-decarboxylated betanins (Table 2).

### 2.1. High-Resolution Mass Spectrometric Study on Mono-Chlorinated Betanin and Its Decarboxylated Derivatives

The fragmentation patterns of **1a**, **2a**, **3a**, **4a**, **5a**, and **6a** were initially obtained by a triple quadrupole mass spectrometry (Table 1) and were confirmed by multi-step ion-trap fragmentation experiments by the high-resolution IT-TOF technique (Table 2, Figure 3 and Figure S2). The LC-IT-TOF analyses in the positive mode yielded high-resolution *m*/*z* values, which supported their identification (Table 2).

Fragmentation of 18-chloro-betanin **1a** (Figure 3 and Table 2) by ion-trap mass spectrometric system gave primarily the characteristic fragment ion profile derived from the neutral losses of glucosyl (*m*/*z* 423 = 585 − 162) as well as HCl (*m*/*z* 387 = 423 − 36) and HCOOH (*m*/*z* 377 = 423 − 46) from generated ion at *m*/*z* 423 (chlorinated betanidin) and subsequently HCl (*m*/*z* 341 = 377 − 36) and HCOOH (*m*/*z* 341 = 387 − 46). Another fragmentation pathway starting from the *m*/*z* 377 ion led to ions observed at *m*/*z* 331 (-HCOOH), *m*/*z* 287 (-CO_2_), *m*/*z* 251 (-HCl), and finally *m*/*z* 150 characteristic for 5,6-dihydroxy-indole. The final ion can appear also by alternative fragmentation reactions from the ion at *m*/*z* 341 through formation of ions observed at *m*/*z* 295 (-HCOOH) and *m*/*z* 251 (-CO_2_) as well as through similar pathway generating ions at *m*/*z* 297 (-CO_2_) and *m*/*z* 253 (-HCOOH). Analogous fragmentation pattern was obtained for deglucosylated derivative, 18-chloro-betanidin **5a** (data not shown).

Fragmentation of 18-chloro-17-decarboxy-betanin **2a** (Table 2 and Figure S2) is based on neutral losses of glucosyl (*m*/*z* 379 = 541 − 162) similar to **1a** as well as HCl (*m*/*z* 343 = 379 − 36) and HCOOH (*m*/*z* 333 = 379 − 46) from generated ion at *m*/*z* 379 (chlorinated 17-decarboxy-betanidin). Further combination of HCOOH and CO_2_ losses from the dechlorinated ion at *m*/*z* 343 (releasing ions at *m*/*z* 299, 297, 255, and 253) as well as neutral losses of the pyridine-based moiety conjugated to nitrogen N-1 concluded the fragmentation down to ions observed at *m*/*z* 150 (5,6-dihydroxy-indole) and 132 (dehydrated 5,6-dihydroxy-indole). These final ions were also observed as a result of a loss of HCOOH (*m*/*z* 287) from the chlorinated ion at *m*/*z* 333 (*m*/*z* 287 = 333 − 46) and subsequent neutral loss of another chloro-pyridinic derivative down to *m*/*z* 150.

Fragmentation profile of 18-chloro-2,17-bidecarboxy-betanin **6a** (Table 2 and Figure S2) is simpler from the profile of **2a** because of the presence of only one carboxylic moiety and it starts with neutral loss of glucosyl (*m*/*z* 335 = 497 − 162) as well as alternate losses of HCl (*m*/*z* 299 = 335 − 36) and HCOOH (*m*/*z* 289 = 335 − 46) at the first stage from the generated ion at *m*/*z* 335 (chlorinated 2,17-bidecarboxy-betanidin) with the subsequent losses of CO_2_ (*m*/*z* 255 = 299 − 44) and HCl (*m*/*z* 253 = 289 − 36) at the second stage. From the ions observed at *m*/*z* 289, 255, and 253, the final fragmentation can proceed down to the ion at *m*/*z* 150 accompanied by a neutral loss of a chloro-pyridinic derivative from the ion of *m*/*z* 289 or dechlorinated pyridinic derivative from the ions of *m*/*z* 253 and 255.

Fragmentation of 18-chloro-15-decarboxy-betanin **3a** (Table 2) is at the first stages similar to **2a** based on neutral losses of glucosyl (*m*/*z* 379 = 541 − 162) as well as HCl (*m*/*z* 343 = 379 − 36) and HCOOH (*m*/*z* 333 = 379 − 46) from generated ion at *m*/*z* 379 (chlorinated 15-decarboxy-betanidin). The difference is based mainly on the lack of the ions observed at *m*/*z* 299 and 255. Instead, combination of HCOOH and CO_2_ losses from the dechlorinated ion at *m*/*z* 343 released ions at *m*/*z* 297, 253, and 251. Further fragmentation concluded on ions at *m*/*z* 150 and 132 as in the case of **2a.** Neutral losses of HCOOH and HCl from the chlorinated ion at *m*/*z* 333 resulted in a formation of ions at *m*/*z* 287 and 297, respectively.

Fragmentation of 18-chloro-2-decarboxy-betanin **4a** (Table 2) at the first stages is similar to **3a** neutral losses of glucosyl (*m*/*z* 379 = 541 − 162) as well as HCl (*m*/*z* 343 = 379 − 36) and HCOOH (*m*/*z* 333 = 379 − 46) from generated ion at *m*/*z* 379 (chlorinated 2-decarboxy-betanidin). The difference is based mainly on the lack the ions observed at *m*/*z* 287 and 251. Combination of HCOOH and CO_2_ losses from the dechlorinated ion at *m*/*z* 343 released ions at *m*/*z* 297 and 253. Further fragmentation concluded on ions at *m*/*z* 150 and 132 as in the cases of **2a** and **3a**.

### 2.2. NMR Structural Elucidation of 18-Chloro-Betanin and Its Decarboxylated Derivatives

For the aim of proving the position of chlorination in betanin **1** and its decarboxylated derivatives **2** and **6**, a preparative semi-synthesis of monochloro-betanin **1a**, monochloro-17-decarboxy-betanin **2a**, and monochloro-2,17-decarboxy-betanin **6a** as well as their analysis by ^1^H and ^13^C two-dimensional NMR techniques were performed. For a chlorination product of betanidin **5**, no attempt of NMR analysis was undertaken because of its low stability.

The results obtained for chlorinated derivatives **1a**, **2a**, and **6a** (Table 3) were compared with NMR data of their respective precursors **1**, **2**, and **6**. In another contribution, we were able to perform the first proton NMR measurement for 18-monochloro-17-decarboxy-betanin **2a** taking advantage of the detection of the additional diagnostic proton at position C-17 [6]. In the precursor **2**, decarboxylation at position C-17 leads primarily to the formation of an individual coupled ^1^H spin system for H-17 and H-18, therefore, a doublet for proton H-17 with a chemical shift of δ 7.50 ppm was observed in the ^1^H spectrum of **2** [6]. The signal observed for proton H-17 characterized by a chemical shift value of δ 7.73 ppm in the form of singlet for 18-chloro-17-decarboxy-betanin **2a** and simultaneously its lack for proton H-18 suggested hydrogen substitution with chlorine atom at carbon C-18.

The results of ^1^H NMR, gCOSY, and gTOCSY analyses obtained for 18-chloro-betanin **1a** in D_2_O (Table 3) enabled to distinguish and characterize several diagnostic, conjugated spin systems characteristic for the structure of betanin (H-2, H-3ab; H-11, H-12; H-14ab, H-15). First was a three-spin system H-15/H-14ab, easily distinguishable in the gCOSY, and gTOCSY spectra as a set of cross-peaks indicating additionally the presence of a carboxyl group at carbon C-15. The H-2/H-3ab system, indicating no decarboxylation at carbon C-2 in the betanin skeleton. The doublets derived from the protons H-11 and H-12 were easily distinguishable by their down- and upfield shifts, respectively. Signals derived from protons H-4 and H-7 appeared as singlets, and a slight shift between them, in the order of 0.08–0.15 ppm, indicated the presence of the hydroxyl group at carbon C-6 of the betanidin fragment. A lack of a signal for H-18 even in the ^1^H NMR spectrum for freshly prepared solution of **1a** (avoiding the relatively fast deuterium exchange) indicated the substitution position by chlorine at carbon C-18.

The dihydroindolic system was indicated by gHSQC correlations of H-2, H-3ab, H-4, and H-7 with their respective carbons. In the dihydropyridinic system, correlations of H-14ab and H-15 with their respective carbons in the HSQC spectra were visible.

Further analysis of data obtained by gHMBC (Table 3, Figure S3) provided a comprehensive structure elucidation owing to a multitude of correlations for the dihydroindolic (H-2 to C-3,8,9,10; H-3ab to C-2,4,5,6,8,9,10; H-4 to C-3,5,6,7,8,9; H-7 to C-5,6,8,9) and dihydropyridinic systems (H-11 to C-2,7,12,14,15; H-12 to C-14,18; H-14ab to C-12,17,18,19; H-15 to C-12,13,14,17,18,19) interconnected by the H-11,12 protons. The positions of all the carbons were confirmed in the ^13^C spectrum of **1a** that additionally indicated position of the carbon C-20 signal (169.7 ppm) from the carboxyl moiety attached to C-17 (Table 3).

Confirmation of the sugar ring system was performed by the gCOSY, gTOCSY, gHSQC, and gHMBC correlations (Table 3). The position of the glycosidic bond at the phenolic carbon C-5 was additionally confirmed by the gHMBC correlation (Figure S3) of the anomeric proton H-1’ with carbon C-5. The *β*-linkage between the aglycone and glucopyranosyl moiety was denoted by the three-bond vicinal proton coupling constant ^3^*J*_1’-2’_ ~ 6–7 Hz.

Analysis of the NOESY spectra (Figure S3) established the typical (*E*)-configuration for C(12)=C(13) and s-*trans* conformation for the dienyl moiety N(1)=C(11)-C(12)=C(13) in **1a** by correlations between protons H-11 with H-7,12,15; as well as H-12 with H-2,3,14ab; and H-14ab with H-7,11,15. Additionally, several NOESY correlations were visible in the sugar ring and between H-3ab with H-2, H-4 as well as the glucosidic proton H-1’ (Figure S3). Above analysis completed the structure identification of 18-chloro-betanin **1a**.

Similar NMR data were obtained for the chlorinated decarboxylated derivatives **2a** and **6a** (Table 3). The presence of the singlet for H-17 at δ 7.73 and 7.63 ppm in **2a** and **6a**, respectively, and simultaneous lack of signal for the proton H-18 indicated the substitution of hydrogen with chlorine at carbon C-18. Additionally, the diagnostic presence of H-17 revealed the upfield shift of C-13 signal (in comparison to **1a**) and the position of the C-18 signal by the HMBC correlations as well as the lack of the carboxyl moiety at carbon C-17.

The additional decarboxylation at carbon C-2 in **6a** (Table 3), which leads to the formation of an individual spin system H-2ab/H-3ab was also observed as in the case of NMR analysis of the precursor 2,17-bidecarboxy-betanin **6**. This was assisted by the appearance of a broad triplet, shifted upwards to δ 4.04 ppm for H-2ab compared to the double doublet H-2 at δ 4.72 ppm in 18-chloro-17-decarboxy-betanin **2a**, as well as the upfield shift of the signal for protons H-3ab. 

Above results confirmed a special position of the attack by HOCl and/or Cl_2_O molecules which is not in the aromatic ring of betanin (typical Cl_2_ target) but in the carbon position C-18 with acidic hydrogen [21].

## 3. Materials and Methods

### 3.1. Reagents

Sodium hypochlorite solution, acetonitrile, ethanol, formic acid, acetone (HPLC grade), methanol (MS grade), H_2_O_2_, NaCl, myeloperoxidase from human leukocytes (MPO), deuterated trifluoroacetic acid and D_2_O were obtained from Sigma Chemical Co. (St. Louis, MO, USA).

### 3.2. Preparation of Betalains from Beta vulgaris L. Extracts

For determination of the ability of certain betalains, and BRE extract as a whole, to react with hypochlorous acid, the following pigments (Figure 1) were derived directly from the BRE extract by semipreparative chromatography: betanin **1**, 17-decarboxy-betanin **2**, 15-decarboxy-betanin **3** [16]. Other pigments present at lower concentrations in the BRE extract (2-decarboxy-betanin **4** and 2,17-bidecarboxy-betanin/-isobetanin **6**/**6’**) were obtained by heating of previously isolated betanin according to already-known procedure [22] as well as by enzymatic deglucosylation (betanidin **5**) [5].

For the direct isolation of the pigments from BRE obtained from FutureCeuticals, Inc. (Momence, IL, USA), 10 g of the extract was dissolved in 10 L of water and was initially purified by ion-exchange chromatography. Obtained fractions were subjected to further semi-preparative HPLC purification. 2-decarboxy-betanin **4** and 2,17-bidecarboxy-betanin/-isobetanin **6**/**6’** were obtained by thermal degradation of aqueous and ethanolic solutions of purified betanin acidified with glacial acetic acid at 85 °C (aqueous solution) and 75 °C (ethanolic solution) in a water bath for 40 min according to previously published procedure [22].

### 3.3. Chromatographic and Mass Spectrometric Determination of the Identity of the Generated Chlorinated Betacyanins in the Chemical (NaOCl) and Enzymatic Systems (MPO)

Chemical chlorination of betacyanins at analytical scale for determination of the identity of the generated chlorinated derivatives over the whole tested physiological and non-physiological pH range was performed by means of NaOCl aqueous solutions (forming the Cl_2_/HOCl/OCl^−^/Cl_2_O system of varying profile of the chlorine entities depending on pH [5]). The reactions were performed in aqueous solutions buffered with 25 mM acetate (pH 3–5) and phosphate (pH 6–8) buffers in 96-well plates of an Infinite 200 microplate reader (Tecan Austria GmbH, Grödig/Salzburg, Austria). The concentration of the stock NaOCl solution was determined spectrophotometrically at pH 12 (ε_292_: 350/M/cm). Just before the measuring step, 20 µL of freshly prepared NaOCl solution from the stock solution was dispensed to each well containing 40 µM dissolved pigment, bringing the volume to 200 µL. The final concentration of the NaOCl solution in the wells was in the range of 40–120 µM for all the tested pigments. The mixture was then shaken for 20 s and the spectra were collected over 5 or 30 min at a temperature of 22 °C by spectrophotometric detection at the wavelength range of 380–600 nm. For the chromatographic analyses, 10 µL samples after 5 min of reaction were injected directly to the LC-DAD-ESI-MS system.

The enzymatic chlorination experiments in the MPO/H_2_O_2_/Cl^−^ system were performed in 25 mM acetate (pH 3–5) and phosphate (pH 6–8) buffers during a reaction catalyzed by 1 µM MPO in the presence of 100 µM H_2_O_2_ and 150 mM NaCl. Just before the measuring step, 20 µL of H_2_O_2_ solution was dispensed to each sample well containing 40 µM betacyanins, bringing the volume to 200 µL. The spectra were collected over 60 min of the experiment. For the chromatographic analyses, each injection to the LC-DAD-ESI-MS system was performed after 60 min of the reaction.

### 3.4. Preparative Chlorination of Betanin and Its Decarboxylated Derivatives

Chlorination of each of the isolated pigments (40 µM) was performed in 1 L aqueous buffered solutions (25 mM acetate, pH 3) by addition of 100 mL NaOCl (100 µM) aqueous solution during mixing by a magnetic stirrer. The concentration of the stock NaOCl solution was determined spectrophotometrically at pH 12 (ε_292_: 350/M/cm). Just before the reaction, the NaOCl solution was freshly prepared from the stock solution and added to the reaction mixture after 15 min. The reaction was completed within few minutes [5] and the measured pH after the reaction was 3. It was not recommended to add an excess of the NaOCl solution in order to avoid accompanying oxidation of the pigments. After 15 min since the beginning of the reaction, the solution was fractionated and purified by flash chromatography and preparative HPLC.

### 3.5. Chromatographic Analysis by LC-DAD-ESI-MS/MS System

For the chromatographic and mass spectrometric analyses, an LCMS-8030 mass spectrometric system (Shimadzu, Kyoto, Japan) was used [5]. Positive ion electrospray mass spectra were recorded on the LC-MS system, which was controlled by LabSolutions software for registration of total ion chromatograms, mass spectra, and ion chromatograms in selected ion monitoring mode (SIM) as well as the fragmentation spectra. The ionization electrospray source (ESI+) was operated at an electrospray voltage of 4.5 kV and capillary temperature at 250 °C, using N_2_ as a gas for the spray. Argon was used as the collision gas for the collision-induced dissociation (CID) experiments. The relative collision energies for MS/MS analyses were set at −35 V in an arbitrary scale.

High resolution mass spectra were recorded using LCMS-IT-TOF mass spectrometer (Shimadzu) equipped with an electrospray ion source (ESI+) and coupled to the HPLC Prominence (Shimadzu). Separation of the compounds was carried out in the gradient system [5] as in the case of the LC-DAD-ESI-MS/MS analyses. Parameters of LCMS-IT-TOF spectrometer were set as follows: curved desolvation line (CDL) and heat block temperature 230 °C, nebulizing gas flow rate 1.5 L/min and capillary voltage 4.5 kV. All mass spectra, including fragmentation mass spectra, were recorded in the positive ion mode with mass range of 100–2000 Da and collision energy between 15 and 50% depending on the respective compound’s structure. The results of high-resolution mass spectrometry experiments (HRMS) were studied using the Formula Predictor within the LCMS Solution software. Only empirical formulae with a mass error below 5 ppm were considered relevant.

### 3.6. NMR Experiments

The NMR spectra were recorded on an Agilent DD2 800 spectrometer (Agilent Technologies, Santa Clara CA, USA) for 7–8 mg of **1a**, **2a**, and **6a** (in non-acidified D_2_O) at 280 K. For samples dissolved in D_2_O, chemical shifts were referenced to TSP (δ_H_ = 0.0 ppm, δ_C_ = 0.0 ppm). All 1D (^1^H) and 2D NMR (gCOSY, gHSQC, gHMBC, TOCSY and NOESY (g = gradient enhanced)) measurements were performed using standard Agilent pulse sequences. The residual water resonance for experiments recorded in D_2_O was suppressed using the low-power presaturation.

## 4. Conclusions

This structural study confirmed the chlorination position in betanin which is not in the aromatic ring (as would be suggested by most of reports) but in the dihydropyridinic moiety at carbon C-18. Therefore, from the aqueous free chlorine equilibrium species: HOCl, OCl^−^, Cl_2_, and Cl_2_O, the most potent chlorinating agents are HOCl and Cl_2_O [17], which we postulated previously [5]. Consequently, the reactions with HOCl and Cl_2_O are proposed according to an electrophilic mechanism based on leaving group ability from Cl^+^ in HOCl (-OH^−^) and in Cl_2_O (-OCl^−^).

## Figures and Tables

**Table 1:** Spectrophotometric and low-resolution mass spectrometric data of the studied betanin, decarboxylated betanins and their derivatives.

No.	Compound	λ_max_ [nm]	*m*/*z* [M + H]^+^	*m*/*z* from MS/MS of [M + H]^+^
**1**	betanin	538	551	389
**1a**	18-chloro-betanin	522	585	423
**2**	17-decarboxy-betanin	505	507	345
**2a**	18-chloro-17-decarboxy-betanin	524	541	379
**3**	15-decarboxy-betanin	528	507	345
**3a**	18-chloro-15-decarboxy-betanin	514	541	379
**4**	2-decarboxy-betanin	533	507	345
**4a**	18-chloro-2-decarboxy-betanin	525	541	379
**5**	betanidin	540	389	345
**5a**	18-chloro-betanidin	528	423	377
**6/6’**	2,17-bidecarboxy-betanin/-isobetanin	507	463	301
**6a**	18-chloro-2,17-bidecarboxy-betanin	519	497	335
**7**	neobetanin	466	549	387; 343; 299

**Table 2:** High-resolution mass spectrometric data obtained by LCMS-IT-TOF for monochlorinated betanin (**1a**) and its decarboxylated derivatives (**2a**, **3a**, **4a**, and **6a**) as well as for their fragmentation ions.

Pigment and Fragmentation Ions ^a^	[M + H]^+^ Molecular Formula	[M + H]^+^ Observed	[M + H]^+^ Predicted	Error [mDa]	Error [ppm]	MS^2^ Ions
*18-chloro-betanin* (**1a**)	C_24_H_26_ClN_2_O_13_	585.1127	585.1118	0.9	1.54	423; 377; 341; 295
nl: -Glc	C_18_H_16_ClN_2_O_8_	423.0599	423.0590	0.9	2.13	387; 377; 341; 331; 297; 295; 287; 253; 251; 150
*18-chloro-17-decarboxy-* *-betanin* (**2a**)	C_23_H_26_ClN_2_O_11_	541.1211	541.1220	−0.9	−1.66	379; 343; 333; 297; 253
nl: -Glc	C_17_H_16_ClN_2_O_6_	379.0687	379.0691	−0.4	−1.06	343; 333; 299; 297; 287; 255; 253; 150; 132
*18-chloro-15-decarboxy-* *-betanin* (**3a**)	C_23_H_26_ClN_2_O_11_	541.1234	541.1220	1.4	2.59	379; 333; 297; 251
nl: -Glc	C_17_H_16_ClN_2_O_6_	379.0680	379.0691	−1.1	−2.90	343; 333; 297; 287; 253; 251; 150; 132
*18-chloro-2-decarboxy-* *-betanin* (**4a**)	C_23_H_26_ClN_2_O_11_	541.1239	541.1220	1.9	3.51	379; 333; 297; 253
nl: -Glc	C_17_H_16_ClN_2_O_6_	379.0697	379.0691	0.6	1.58	343; 333; 297; 253; 150; 132
*18-chloro-2,17-bidecarboxy--betanin* (**6a**)	C_22_H_26_ClN_2_O_9_	497.1339	497.1321	1.8	3.62	335; 299; 253
nl: -Glc	C_16_H_16_ClN_2_O_4_	335.0785	335.0793	−0.8	−2.39	299; 289; 255; 253; 150

^a^ nl—neutral losses from [M + H]^+^.

**Table 3:** The NMR data of analyzed 18-chloro-betanin **1a**, 18-chloro-17-decarboxy-betanin **2a** and 18-chloro-2,17-bidecarboxy-betanin **6a**. Important HMBC and NOESY correlations for **1a** are depicted in Figure S3.

	18-Chloro-Betanin (1a)		18-Chloro-17-Decarboxy-Betanin (2a)		18-Chloro-2,17-Bidecarboxy-Betanin (6a)	
No.	^1^H NMR ^a^	^13^C NMR ^b,c^	^1^H NMR ^a^	^13^C NMR ^b,c^	^1^H NMR ^a^	^13^C NMR ^b,c^
**2 or 2a/b**	4.90, *dd*, 2.1; 8.4	68.0	4.72, *dd*, 3.1, 10.5	67.8	4.04, *bt*	53.1
**3a/b**	3.60 (overlap) 3.19, *dd*, 2.5; 16.4	36.2	3.61 (overlap) 3.18, *dd*, 3.4, 16.9	35.6	3.09, *bt*	29.5
**4**	7.12, *s*	116.7	7.09, *s*	115.9	6.97, *s*	115.9
**5**		147.1		147.2		146.1
**6**		149.4		148.7		147.8
**7**	7.00, *s*	102.9	7.02, *s*	102.6	6.82, *s*	102.5
**8**		140.2		139.7		138.6
**9**		127.3		126.8		128.5
**10**		178.4		177.9		-
**11**	8.29, *d*, 12.4	147.6	8.21, *d*, 12.5	146.9	8.02, *d*, 11.9	146.7
**12**	5.97, *d*, 12.4	105.7	5.83, *d*, 12.6	104.4	5.83, *d*, 12.0	104.7
**13**		163.8		156.8		156.1
**14a/b**	3.37, *bm*	30.8	3.22, *bm*,	30.2	3.18, *bm*	30.4
**15a/b**	4.48, *t*, 7.5	55.7	4.22, *t*, 8.5	56.3	4.22, *t*, 7.1	56.7
**17**		158.9	7.73, *s*	156.3	7.65, *s*	156.2
**18**		105.6		108.2		109.0
**19**		176.7		177.1		177.8
**20**		169.7 ^d^		-		-
**1’**	5.05, *d*, 6.8	104.5	5.04, *d*, 7.2	104.2	5.05, *d*, 6.9	103.9
**2’**	3.59 (overlap)	79.2	3.56 (overlap)	79.1	3.58 (overlap)	79.2
**3’**	3.52	72.3	3.49	72.2	3.51	72.6
**4’**	3.61 (overlap)	75.8	3.58 (overlap)	75.9	3.55 (overlap)	75.6
**5’**	3.61 (overlap)	78.6	3.59 (overlap)	78.4	3.60 (overlap)	78.4
**6’a/b**	3.92, *dd*, 2.1, 12.5 3.77, *dd*, 5.4, 12.5	63.4	3.92, *dd*, 2.2, 12.5 3.76, *dd*, 5.4, 12.6	63.3	3.94, *dd*, 2,6; 12.3 3.76, *dd*, 5.3; 12.3	63.6

^a 1^H NMR *δ* [ppm], mult, *J* [Hz]. ^b 13^C NMR *δ* [ppm]. ^c 13^C chemical shifts were derived from gHSQC and gHMBC. ^d^ Chemical shift obtained from a ^13^C spectrum.

## Supplementary Materials

The supplementary materials are available online.

## References

[1] Khan M.I., Giridhar P. (2015). Plant betalains: Chemistry and biochemistry. Phytochemistry.

[2] Azeredo H.M.C. (2009). Betalains: Properties, sources, applications, and stability—A review. Int. J. Food Sci. Technol..

[3] Gandía-Herrero F., Escribano J., García-Carmona F. (2016). Biological activities of plant pigments betalains. Crit. Rev. Food Sci..

[4] Khan M.I. (2015). Plant betalains: Safety, antioxidant activity, clinical efficacy, and bioavailability. Compr. Rev. Food Sci. Food Saf..

[5] Wybraniec S., Starzak K., Pietrzkowski Z. (2016). Chlorination of betacyanins in several hypochlorous acid systems. J. Agric. Food Chem..

[6] Wybraniec S., Starzak K., Szneler E., Pietrzkowski Z. (2016). Separation of chlorinated diastereomers of decarboxy-betacyanins in myeloperoxidase catalyzed chlorinated *Beta vulgaris* L. extract. J. Chromatogr. B.

[7] Pietrzkowski Z., Nemzer B., Michałowski T., Wybraniec S. (2010). Influence of betalain-rich extract on reduction of discomfort associated with osteoarthritis. New Med..

[8] Pietrzkowski Z., Argumedo R., Shu C., Nemzer B., Wybraniec S., Reyes-Izquierdo T. (2014). Betalain-rich red beet concentrate improves reduced knee discomfort and joint function: A double blind, placebo-controlled pilot clinical study. Nutr. Diet. Suppl..

[9] Kettle A.J., Albrett A.M., Chapman A.L., Dickerhof N., Forbes L.V., Khalilova I., Turner R. (2014). Measuring chlorine bleach in biology and medicine. Biochim. Biophys. Acta Gen. Subj..

[10] Klebanoff S.J. (2005). Myeloperoxidase: Friend and foe. J. Leukoc. Biol..

[11] Zhang R., Song B., Yuan J. (2018). Bioanalytical methods for hypochlorous acid detection: Recent advances and challenges. Trends Anal. Chem..

[12] Albrich J.M., McCarthy C.A., Hurst J.K. (1981). Biological reactivity of hypochlorous acid: Implications for microbicidal mechanisms of leukocyte myeloperoxidase. Proc. Natl. Acad. Sci. USA.

[13] Dennis W.H., Olivieri V.O., Krusé C.W. (1979). Reaction of uracil with hypochlorous acid. Water Res..

[14] Visser M.C.M., Winterbourn C.C. (1991). Oxidative damage to fibronectin. I. The effects of the neutrophil myeloperoxidase system and HOCl. Arch. Biochem. Biophys..

[15] Carr A.C., Vissers M.C.M., Domigan N.M., Winterbourn C.C. (1997). Modification of red cell membrane lipids by hypochlorous acid and haemolysis by preformed lipid chlorohydrins. Redox Rep..

[16] Nemzer B., Pietrzkowski Z., Spórna A., Stalica P., Thresher W., Michałowski T., Wybraniec S. (2011). Betalainic and nutritional profiles of pigment-enriched red beet root (*Beta vulgaris* L.) dried extracts. Food Chem..

[17] Sivey J.D., McCullough C.E., Roberts A.L. (2010). Chlorine monoxide (Cl_2_O) and molecular chlorine (Cl_2_) as active chlorinating agents in reaction of dimethenamid with aqueous free chlorine. Environ. Sci. Technol..

[18] Agrawal S., Ingle N., Maity U., Jasra R.V., Munshi P. (2018). Effect of Aqueous HCl with Dissolved Chlorine on Certain Corrosion-Resistant Polymers. ACS Omega.

[19] Lau S.S., Reber K.P., Roberts A.L. (2019). Aqueous Chlorination Kinetics of Cyclic Alkenes-Is HOCl the Only Chlorinating Agent that Matters?. Environ. Sci. Technol..

[20] Swain C.G., Crist D.R. (1972). Mechanisms of chlorination by hypochlorous acid. The last of chlorinium ion, Cl^+^. J. Am. Chem. Soc..

[21] Strack D., Steglich W., Wray V., Dey P.M., Harborne J.B., Waterman P.G. (1993). Betalains. Methods in Plant Biochemistry.

[22] Wybraniec S. (2005). Formation of decarboxylated betacyanins in heated purified betacyanin fractions from red beet root (*Beta vulgaris* L.) monitored by LC−MS/MS. J. Agric. Food Chem..

