# Supplementary material for: Structural Study on Hypochlorous Acid-Mediated Chlorination of Betanin and Its Decarboxylated Derivatives from an Anti-Inflammatory Beta vulgaris L. Extract
Source: Molecules. 2020 Jan 16;25(2):378. doi: 10.3390/molecules25020378 (PMC7024289; doi:10.3390/molecules25020378)
Supplement: Supplementary file 1 [file molecules-25-00378-s001.pdf]

# Supplementary Materials

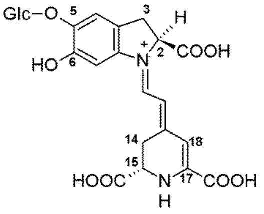


betanin **1**; *m/z* 551

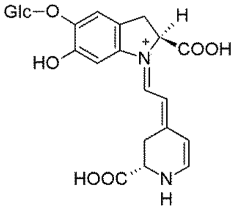


17-decarboxy-betanin **2**;
*m/z* 507

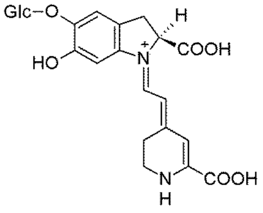


15-decarboxy-betanin **3**;
*m/z* 507

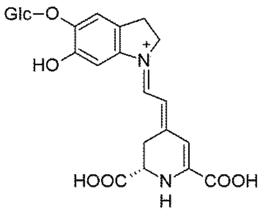


2-decarboxy-betanin **4**;
*m/z* 507

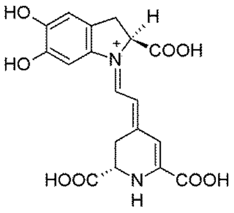


betanidin **5**; *m/z* 389

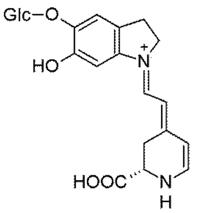


2,17-bidecarboxy-betanin **6**;
*m/z* 463

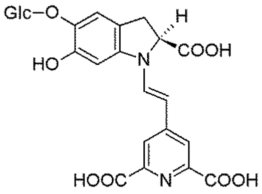


neobetanin **7**; *m/z* 549

**Figure 1.** Chemical structures of betanin **1**, its derivatives (**2-4**, **6-8**) and betanidin **5** present in the BRE extract subjected to studies on chlorination.

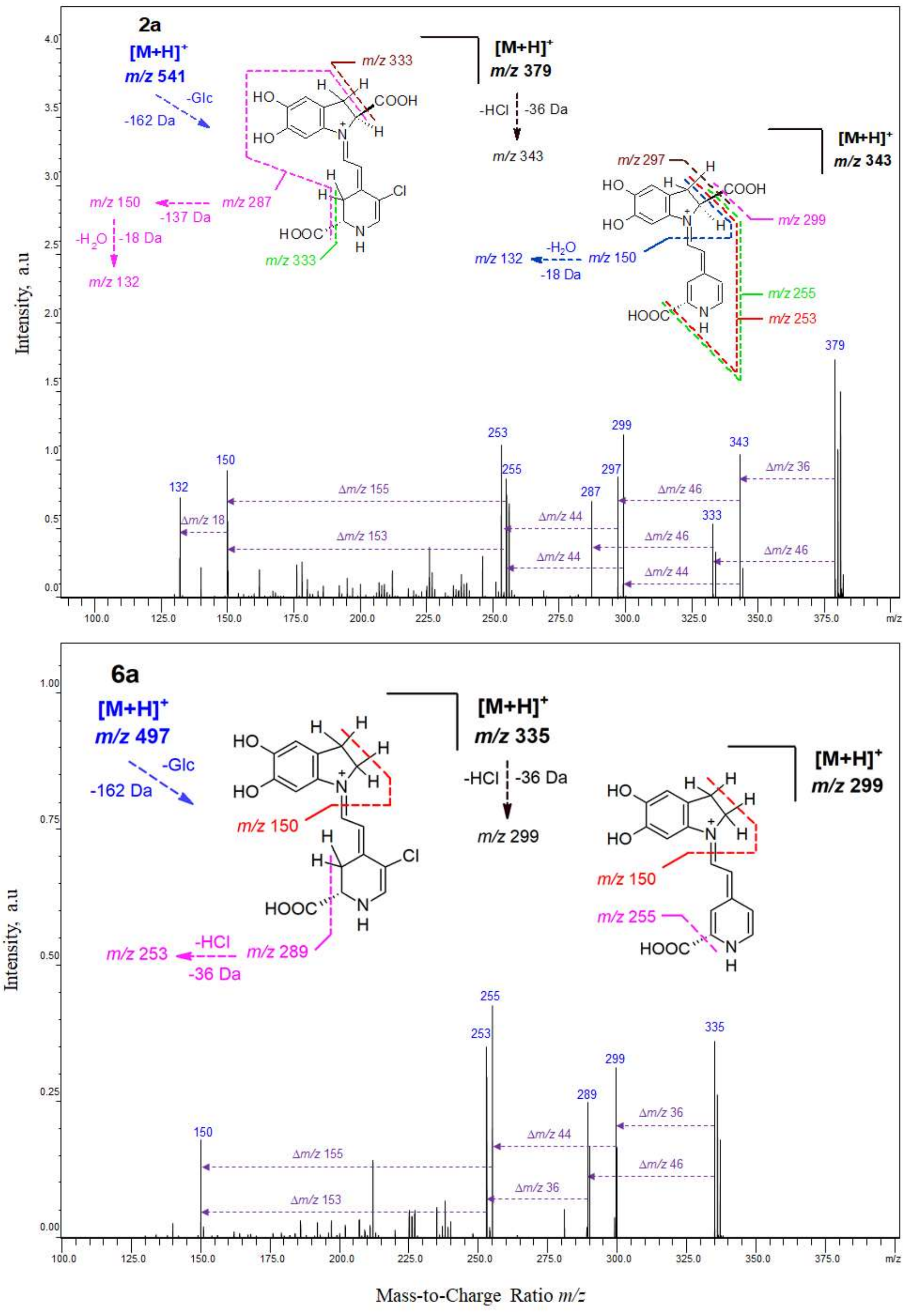


**Figure 2.** The HRMS fragmentation spectra obtained by IT-TOF for chlorinated decarboxylated derivatives 18-chloro-17-decarboxy-betanin **2a** and 18-chloro-2,17-bidecarboxy-betanin **6a** as well as the fragmentation pathways for their deglucosylated protonated ions.

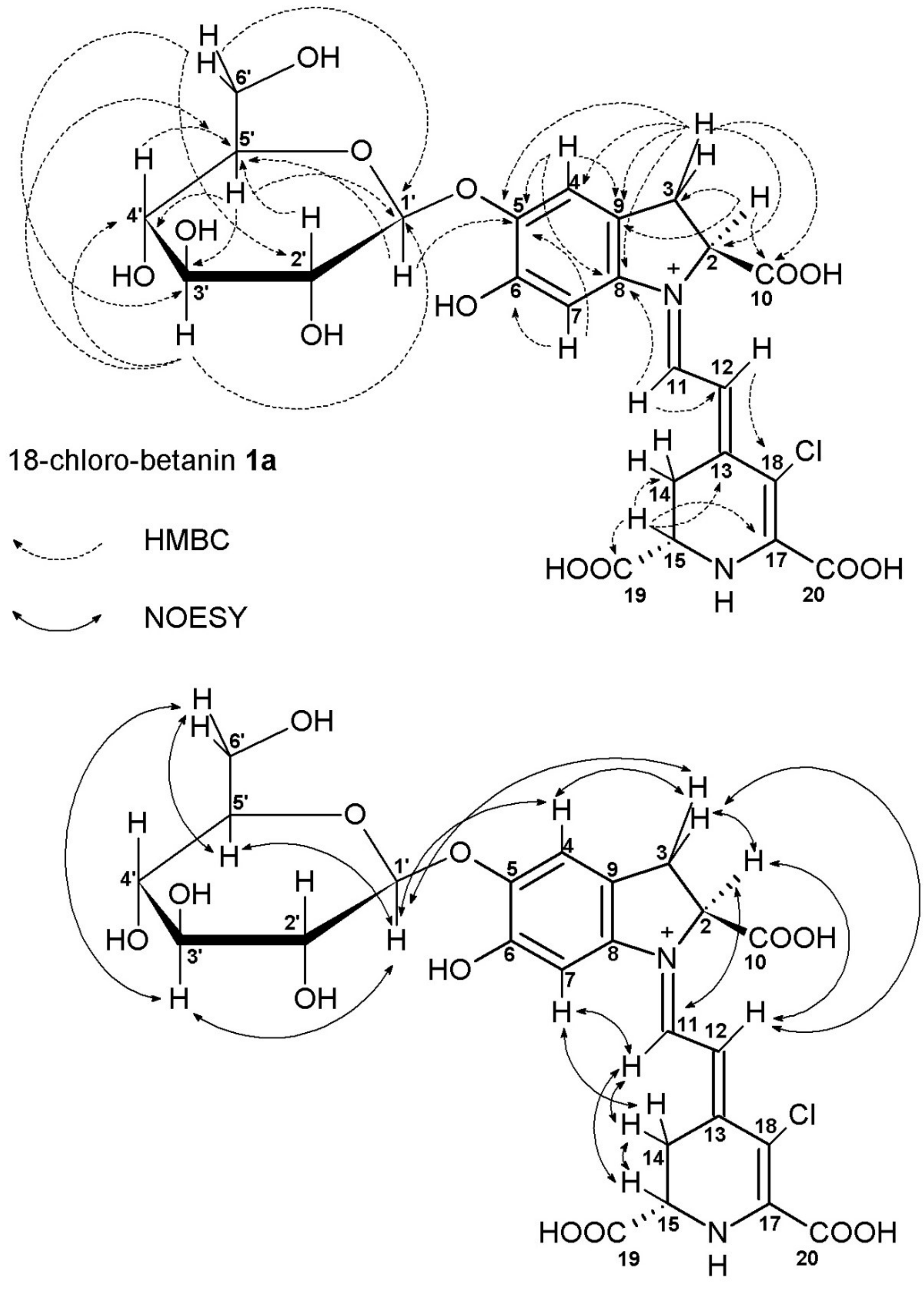


**Figure 3.** Important HMBC and NOESY NMR correlations in the structurally elucidated 18-chloro-betanin **1a**.
